# Ameliorating Effects of Coriander on Gastrocnemius Muscles Undergoing Precachexia in a Rat Model of Rheumatoid Arthritis: A Proteomics Analysis

**DOI:** 10.3390/nu13114041

**Published:** 2021-11-12

**Authors:** Huijuan Jia, Ya Wen, Wanping Aw, Kenji Saito, Hisanori Kato

**Affiliations:** 1 Health Nutrition, Department of Applied Biological Chemistry, The University of Tokyo, 1-1-1, Yayoi, Bunkyo-ku, Tokyo 113-8657, Japan; ya.wen@ki.se (Y.W.); wanping@sfc.keio.ac.jp (W.A.); skkj774@gmail.com (K.S.); 2 Department of Physiology and Pharmacology, Karolinska Institutet, Bioclinicum, J8:30, SE-171 77 Stockholm, Sweden; 3 Institute for Advanced Biosciences, Keio University, 246-2, Mizukami, Kakuganji, Tsuruoka, Yamagata 997-0052, Japan

**Keywords:** coriander, rheumatoid arthritis, precachexia, gastrocnemius skeletal muscle, proteomics

## Abstract

Coriander is a commonly used vegetable, spice, and folk medicine, possessing both nutritional and medicinal properties. Up to two-thirds of patients with rheumatoid arthritis (RA) exhibit loss of body mass, predominately skeletal muscle mass, a process called rheumatoid cachexia, and this has major effects of the quality of life of patients. Owing to a lack of effective treatments, the initial stage of cachexia has been proposed as an important period for prevention and decreasing pathogenesis. In the current study, we found that cachexia-like molecular disorders and muscle weight loss were in progress in gastrocnemius muscle after only 5 days of RA induction in rats, although rheumatoid cachexia symptoms have been reported occurring approximately 45 days after RA induction. Oral administration of coriander slightly restored muscle loss. Moreover, iTRAQ-based quantitative proteomics revealed that coriander treatment could partially restore the molecular derangements induced by RA, including impaired carbon metabolism, deteriorated mitochondrial function (tricarboxylic acid cycle and oxidative phosphorylation), and myofiber-type alterations. Therefore, coriander could be a promising functional food and/or complementary therapy for patients with RA against cachexia.

## 1. Introduction

Coriander (*Coriandrum sativum* L.) is a widely distributed Apiaceae family plant. This annual herbaceous plant is extensively cultivated in North Africa, Central Europe, and Asia, although it originated in the Mediterranean region [1]. All parts of coriander are edible, regardless of the distinct and unique flavor. The fresh green leaves are commonly used in Thai and Vietnamese cuisine. The roots are used in Asian cuisines, and the stems are chopped and used in soups and stews [2]. Besides being widely used as a spice and vegetable in cuisines from various regions and countries, coriander also has a long history of being used as a traditional remedy for treating various disorders in different civilizations. In the northern areas of Pakistan, coriander has been used as a folk medicine to treat digestive system disorders, such as diarrhea, stomach pain, and vomiting [3]. In the Indian traditional medicinal system, coriander has been used for the treatment of disorders of the urinary, respiratory, and digestive systems [4].

Rheumatoid arthritis (RA) is a chronic and systemic inflammatory disease affecting approximately 1% of the population worldwide, with greater incidence among elders, women, and smokers [5,6,7]. In addition to the major articular manifestations, including joint pain, swelling, and stiffness, patients with RA also suffer from cachexia, which is prevalent in up to two-thirds of patients [8]. Cachexia is an involuntary loss of body mass, predominately skeletal muscle, accompanied by deterioration of physical performance, disruption of metabolism, and reduction in quality of life [5,9,10,11]. In a rat model, rheumatoid cachexia was observed around 45 days after RA induction [12].

In chronic and systemic inflammation diseases, cachexia occurs via several stages, including “precachexia”, “cachexia”, and “refractory cachexia” [13]. Owing to the lack of effective treatments for cachexia, recent research has focused on precachexia, i.e., the initial phase of cachexia, in which interventions may slow down the pathogenetic process and prevent muscle wasting [13,14]. In the most studied precachexia in cancer, patients normally present with minor weight loss (≤5%), anorexia, metabolic changes, and systemic inflammation [15]. However, in patients with RA, manifestations are barely perceptible (e.g., weight loss and decreased appetite) during the pathogenetic process toward rheumatoid cachexia, making it difficult to identify and diagnose patients during this transitional period [8,9]. Therefore, studies of molecular alterations in these patients may facilitate the investigation of the initial phase of rheumatoid cachexia.

With the development of liquid chromatography tandem mass spectrometry (LC-MS/MS) technologies, proteomics approaches have become prevalent tools for universal detection of molecular alterations and interactions in specific biological samples. Thus, proteomics studies can be applied for elucidation of the etiology and pathogenesis of certain diseases, identification of disease biomarkers, and discovery and efficacy assessment of novel drugs and functional foods [16,17,18].

Therefore, in this study, we investigated molecular alterations in the gastrocnemius muscle after five days of RA induction in rats and explored and evaluated the potential effects of coriander treatment via a proteomics approach. Our findings provide insights into the potential applications of coriander as a functional food and/or complementary therapy for preventing and slowing the pathogenesis of rheumatoid cachexia.

## 2. Materials and Methods

### 2.1. Animal Experiment

After 3 days of acclimatization with an American Institute of Nutrition (AIN)-93G powdered diet (see ingredients composition in Supplementary Table S1), 8-week-old male Wistar rats purchased from Charles River Japan (Tokyo, Japan) were divided into 3 groups: control group (CON; *n* = 6), RA group, with RA induction by injection of complete Freund’s adjuvant (CFA; Sigma-Aldrich Co., St Louis, MO, USA; *n* = 7), and COR group, with RA induction with coriander treatment (*n* = 7). Rats from the CON and RA groups were fed an AIN-93G diet, whereas rats from the COR group were treated with an extra 2% coriander leaf fine powder (S&B Foods Inc., Tokyo, Japan; Supplementary Table S1). After 14 days of pre-feeding, RA was induced in rats from the RA and COR groups via an intradermal injection of 0.1 mL CFA into the sub-plantar surface of the left hind paw using a 26-gauge needle, according to the Protocol for Adjuvant-Induced Arthritis (AIA) in Rats (Chondrex, Inc., Redmond, WA, USA). Rats from the CON group received a vehicle (olive oil) injection as a normal control. Rats were housed individually in cages and allowed free access to diet and water. Maintenance and treatment of rats were performed following the guidelines of the Animal Care and Use Committee of the University of Tokyo.

### 2.2. Measurement of Physiological Index and RA Biomarkers

Body weight and food intake for each rat were recorded daily after CFA injection. Computed tomography (CT) scanning was conducted using 1 mm slices from tiptoe to heel bone to measure the lower extremity volume on days 0, 2, and 4 after CFA injection using a LaTheta in vivo Micro-CT scanner (Hitachi Aloka Medical, Tokyo, Japan). Blood samples from the tail vein were collected on days 1, 3, and 5. Serum was obtained by centrifuging blood at 1000× *g* for 15 min at 4 °C after blood collection. Enzyme-linked immunosorbent assay kits (Thermo Fisher Scientific Inc., Rockford, IL, USA; R&D Systems Inc., Minneapolis, MN, USA) were used to measure the concentrations of serum tumor necrosis factor alpha (TNFα) and matrix metallopeptidase 3 (MMP3) as biomarkers of systemic inflammation and RA [19,20], respectively. On day 5 after CFA injection, the animals were weighed and sacrificed after 16 h of fasting. The gastrocnemius muscle tissues were dissected, weighed, and stored at −80 °C prior to use.

### 2.3. Protein Preparation and Isobaric Tag for Relative and Absolute Quantitation (iTRAQ)-Based Quantitative Proteomics Analysis

Total protein from gastrocnemius muscles was extracted using lysis buffer following the manufacturer’s instructions (AB SCIEX, Framingham, MA, USA) and was then subjected to centrifugation at 12,000× *g* for 30 min at 4 °C. Protein concentrations were measured using Bradford assays (Bio-Rad Laboratories, Hercules, CA, USA). Protein samples for each group were pooled together (100 µg) before being treated with cysteine blocking and digestion and were then labeled with iTRAQ tags as follows: CON, 114 tag; RA, 115 tag; COR, 116 tag, using a 4-plex iTRAQ labeling kit (AB SCIEX), according to the manufacturer’s recommendations. The reconstituted samples (50 µL) were subjected to nano-LC-MS/MS analysis (AB SCIEX).

### 2.4. Bioinformatics Analyses

ProteinPilot software (AB SCIEX) was used to identify proteins and calculate protein expression levels by comparing with in silico peptide data. Proteins with absolute fold change more than 1.20 and *p*-value < 0.05 were regarded as differentially expressed proteins (DEPs). Subsequently, a dataset of DEPs with protein IDs from the UniProt-rat database and corresponding abundance changes was subject to Kyoto Encyclopedia of Genes and Genomes (KEGG) pathway enrichment analyses using ClusterProfiler [21]. The terms from KEGG enrichment analyses were considered significantly enriched when the adjusted *p*-value was less than 0.05. Fold change information for each DEP was added to our enrichment analyses, reflected as the z-score [22,23], which is an easily calculated value for prediction of the status of the enriched KEGG pathway. The z-score was calculated as follows:

$$zscore=\frac{\left(up-down\right)}{\sqrt{count}}$$

where *count* is the number of genes assigned to an enriched term, and *up* and *down* are the numbers of assigned genes upregulated or downregulated in the data, respectively. This dataset was also subject to Ingenuity Pathway Analysis (IPA) [23] for further confirmation.

### 2.5. Statistical Analyses

All data are presented as means ± standard errors. Differences among three groups were examined by one-way ANOVA followed by Tukey’s test for statistical significance. Results with a *p*-value less than 0.05 indicated statistical significance.

## 3. Results

### 3.1. Assessment of the Physiological Index and Biomarkers of RA

**Figure 1:**
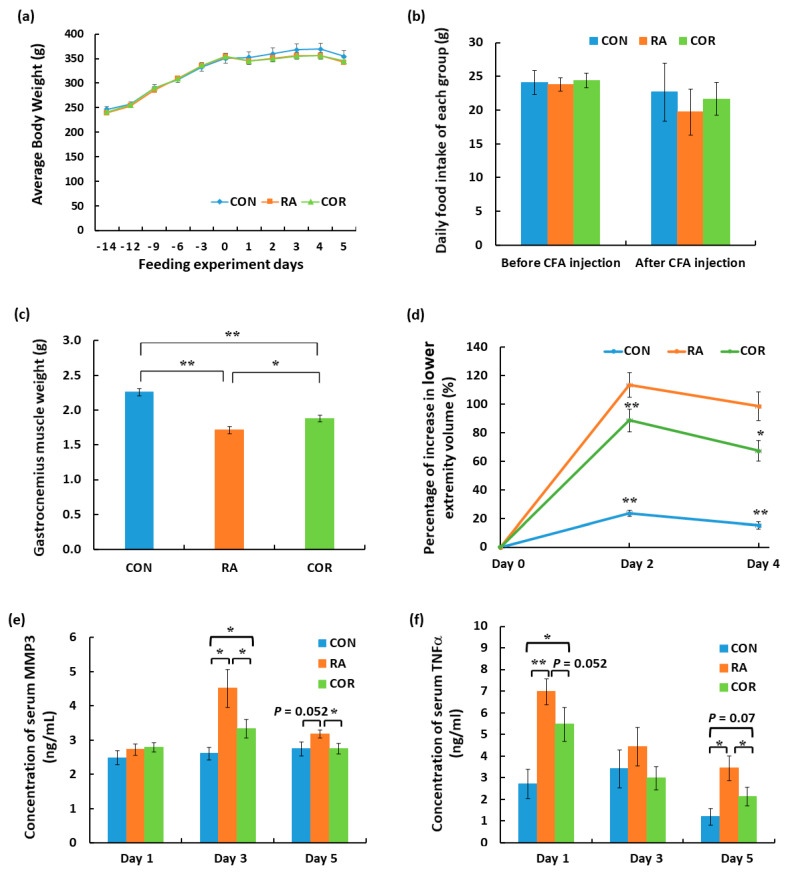
Physiological index and measurement of biomarkers of rheumatoid arthritis (RA) in each group. (**a**) Average body weight, (**b**) daily food intake of each individual in each group, (**c**) gastrocnemius muscle weight, (**d**) percentage of increase in lower extremity volume, and (**e**) MMP3 and (**f**) TNFα serum levels. CON: negative control group; RA: rats with RA induction; COR: rats with RA induction and coriander treatment. Data were expressed as means ± standard errors. ** *p* < 0.01, * *p* < 0.05.

Two physiological indexes were assessed, including body weight and food intake. As shown in Figure 1a,b, there were no significant differences in body weight and food intake among experimental groups during the 14 days of pre-feeding and 5 days of RA induction. However, a significant loss in gastrocnemius muscle weight was observed in the RA group, whereas modest but significant amelioration was observed in the COR group (Figure 1c). To determine the swelling degree of the lower extremities induced by RA, we conducted CT scans at three time points. On days 2 and 4 after CFA injection, there was a significant increase in lower extremity volume in the RA group compared to that in the CON group, whereas the COR group showed a significant decrease in lower extremity volume compared to that in the RA group (Figure 1d). Serum protein levels of MMP3 and TNFα were also evaluated in each group. Notably, on day 3 after CFA injection, MMP3 levels were significantly higher in the RA group than in the CON group, whereas a significant decrease in serum MMP3 was observed in the COR group compared to that in the RA group. This trend was maintained until day 5 after CFA injection (Figure 1e). On the other hand, on days 1 and 5 after CFA injection, TNFα protein levels were significantly higher in the RA group than in the CON group, whereas a significant decrease in serum TNFα level was observed in the COR group compared with that in the RA group (Figure 1f). These results were generally consistent with the description in the Protocol for AIA in Rats (Chondrex, Inc., MA, USA), in which severe and acute inflammation peaks were observed within 3–4 days after CFA injection into the rear paw.

### 3.2. Identification of DEPs

**Figure 2:**
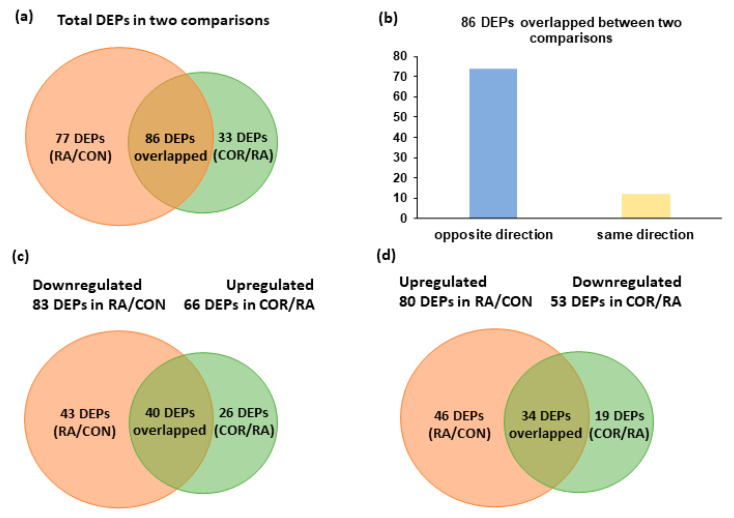
Differentially expressed proteins (DEPs) in the RA/CON and COR/RA comparison. The Venn diagrams show summaries of (**a**) DEPs in both comparisons, (**b**) overlapped DEPs in both comparisons, (**c**) DEPs with downregulated expression in the RA/CON comparison but with upregulated expression in the COR/RA comparison, and (**d**) DEPs with upregulated expression in the RA/CON comparison but with downregulated expression in the COR/RA comparison.

In total, 163 proteins were identified as DEPs between the RA group and the CON group. Of these, 80 DEPs were increased, whereas 83 DEPs were decreased following CFA-induced RA. Moreover, 119 proteins were identified as DEPs between the COR group and the RA group. Of these, 66 DEPs showed increased protein levels, whereas 53 DEPs showed decreased protein levels in RA rats with coriander treatment. Furthermore, 86 of these DEPs were identified in both the RA/CON and COR/RA comparisons (Figure 2a). Among which, 74 DEPs showed opposite directions, whereas 12 DEPs showed the same direction (Figure 2b). Forty DEPs were decreased in the RA/CON comparison but increased in the COR/RA comparison (Figure 2c), whereas thirty-four DEPs were increased in the RA/CON comparison but decreased in the COR/RA comparison (Figure 2d). These results indicated that coriander treatment showed the opposite regulatory effects on 74 DEPs (approximately 46.0%) induced by RA.

### 3.3. KEGG Pathway Analyses

**Figure 3:**
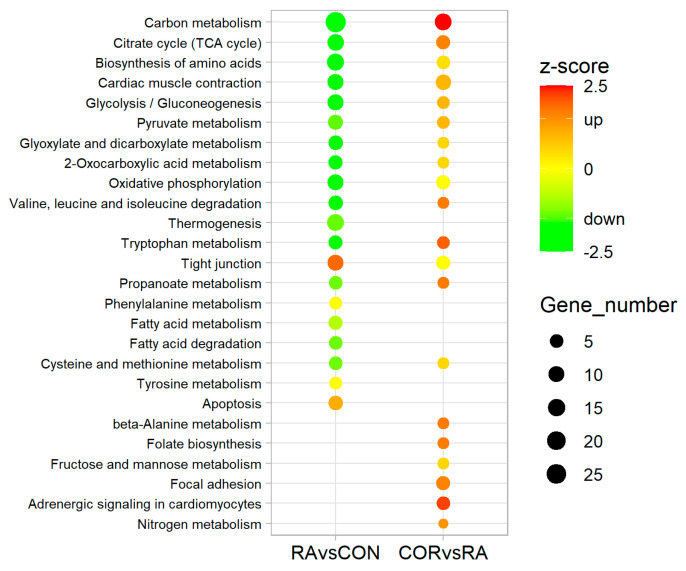
Comparison of enriched KEGG pathways between the RA/CON and COR/RA comparisons. RA: rheumatoid arthritis induction; COR: coriander treatment. Pathways were selected and ranked by *p*-value.

The 20 top-ranked KEGG pathways enriched by the DEPs in the RA/CON comparison are shown in the left column of Figure 3, with the corresponding pathways of the COR/RA comparison shown in the right column. There were many overlapped pathways between the two comparisons, but with opposite statuses.

**Figure 4:**
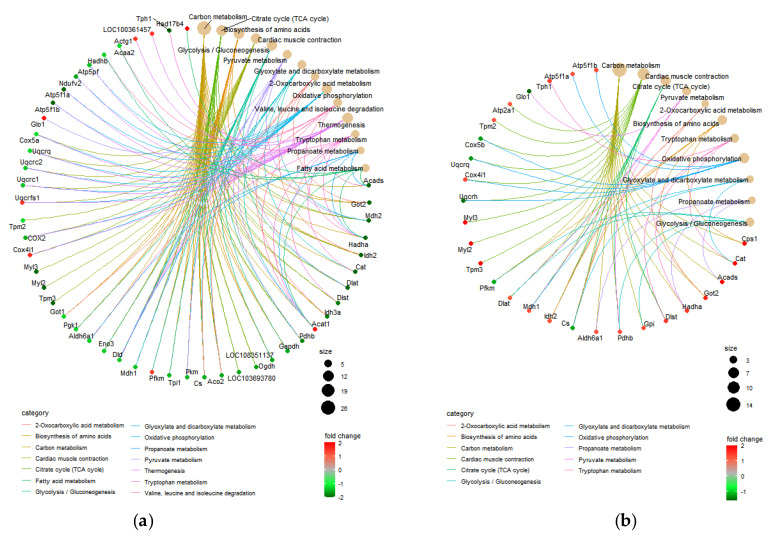
Chord diagram of the most relevant KEGG pathways and involved DEPs in (**a**) the RA/CON comparison and (**b**) the COR/RA comparison.

In the RA/CON comparison, the majority of the KEGG pathways with very low negative z-scores indicated that the corresponding involved DEPs were mostly decreased, suggesting dramatic inhibition of these pathways. For example, RA induction may result in inhibition of pathways related to energy production and metabolism, including “carbon metabolism” (z-score: −4.31), “citrate cycle (TCA cycle)” (z-score: −3.46; Supplementary Figure S1), “glycolysis/gluconeogenesis” (z-score: −2.53; Supplementary Figure S2), and “oxidative phosphorylation” (z-score: −2.11; Supplementary Figure S3). The fold change information for the involved DEPs in the corresponding pathways is shown in Figure 4a.

In the COR/RA comparison, the majority of KEGG pathways with positive z-scores indicated that the involved DEPs were mostly increased, suggesting activation of these pathways. The fold change information of the involved DEPs for the corresponding pathways is shown in Figure 4b, and Supplementary Figures S1–S3. These results suggested that after coriander administration, the abnormalities in molecular pathways induced by RA would be partially restored.

### 3.4. IPA

**Figure 5:**
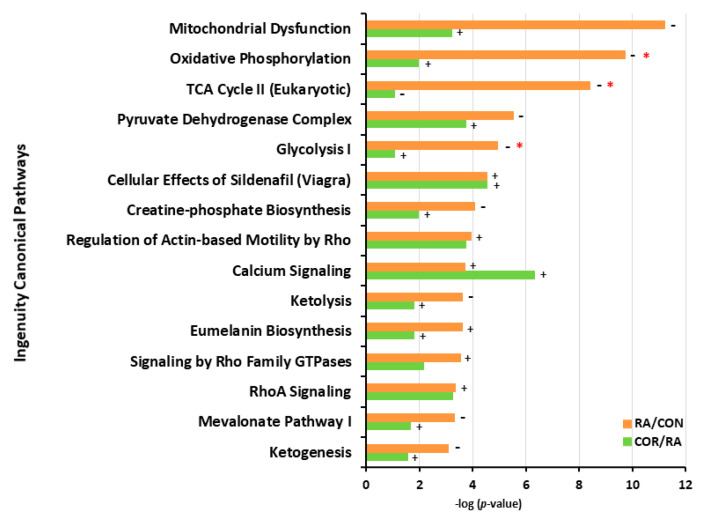
The most significantly altered pathways induced by RA and the effects of coriander treatment. The top 15 canonical pathways in the RA/CON comparison are presented in orange color, whereas the same pathways involved in the COR/RA comparison are presented in green color. “+” indicates that the pathway was predicted to be activated, whereas “–” indicates that the pathway was predicted to be inhibited. “*” in red color represents credible (absolute z-score > 2). −log (*p*-value) > 1.3 represents *p*-value < 0.05.

**Figure 6:**
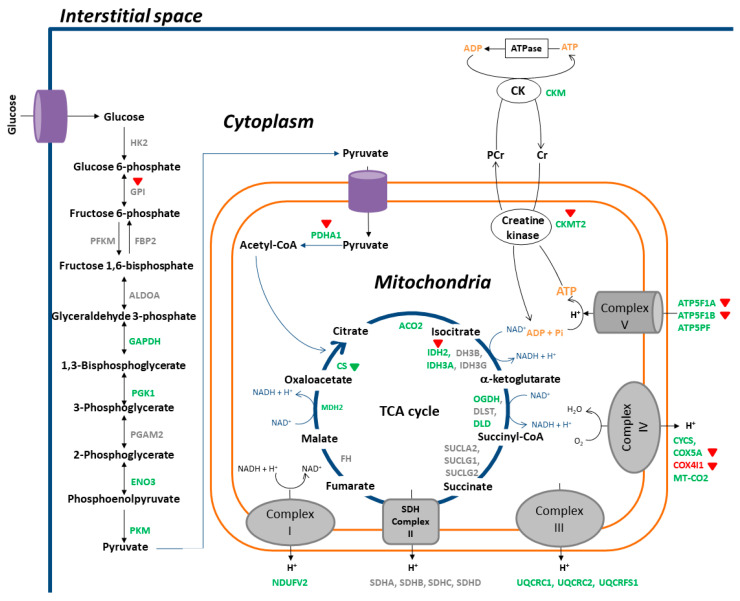
A schematic representation of molecular alterations involved in energy metabolism occurring in gastrocnemius muscles affected by RA and the effects of coriander treatment. Proteins with no significant change or no detection are in gray color. Proteins that were downregulated by RA are in green color, whereas these were upregulated are in red color. Triangles in red color indicate the upregulating effects of coriander, whereas triangles in green color indicate the downregulating effects of coriander.

Based on IPA, the 15 most relevant canonical pathways in the RA/CON comparison are shown in Figure 5. Of these, “mitochondria dysfunction” was regarded as the most relevant canonical pathway that was significantly affected by RA. Other canonical pathways, such as “oxidative phosphorylation”, “TCA cycle”, and “glycolysis”, were also significantly affected by RA. Coriander treatment had the opposite regulatory effects on many of the most relevant canonical pathways, such as “mitochondrial dysfunction”, “oxidative phosphorylation”, “glycolysis”, “pyruvate dehydrogenase complex”, and “creatine-phosphate biosynthesis”. As shown in Figure 6, decreased protein levels were detected in DEPs participating in “oxidative phosphorylation”, including NADH-ubiquinone oxidoreductase subunit (24 kDa, NDUFV2), cytochrome b-c1 complex subunit 1 (UQCRC1), cytochrome b-c1 complex subunit 2 (UQCRC2), cytochrome c (CYCS), cytochrome c oxidase subunit 5A (COX5A), cytochrome c oxidase subunit 2 (MT-CO2), ATP synthase F1 subunit alpha (ATP5F1A), ATP synthase F1 subunit beta (ATP5F1B), and ATP synthase-coupling factor 6 (ATP5PF). Decreased protein levels were also detected in DEPs related to the TCA cycle, including citrate synthase (CS), aconitate hydratase (ACO2), isocitrate dehydrogenase (NADP) (IDH2), isocitrate dehydrogenase (NAD) subunit alpha (IDH3A), 2-oxoglutarate dehydrogenase (OGDH), dihydrolipoyl dehydrogenase (DLD), and malate dehydrogenase (MDH2). Furthermore, decreased protein levels were observed in DEPs participating in glycolysis, including glyceraldehyde-3-phosphate dehydrogenase (GAPDH), phosphoglycerate kinase (PGK1), beta-enolase (ENO3), pyruvate kinase (PKM), and pyruvate dehydrogenase E1 component subunit alpha (PDHA1). In addition, creatine kinase M-type (CKM) and creatine kinase S-type (CKMT2) in the creatine-phosphate biosynthesis pathway were downregulated by RA. Coriander treatment exhibited opposite regulatory effects on several DEPs involved in these biofunctions and pathways in RA rats, including glucose-6-phosphate isomerase (GPI), PDHA1, IDH2, ATP5F1A, ATP5F1B, and CKMT2. These results suggested that RA downregulated pathways related to metabolism, which could be partially restored by coriander treatment. These results were similar to the KEGG pathway analysis.

**Figure 7:**
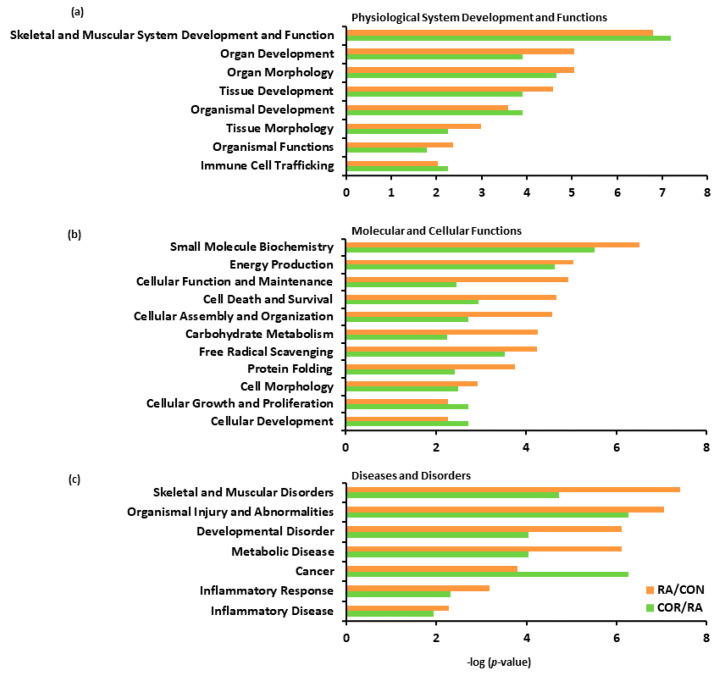
The most relevant biofunctions affected by RA and the effects of coriander treatment. Biofunctions were classified into three primary categories: (**a**) Physiological System Development and Functions, (**b**) Molecular and Cellular Functions, and (**c**) Diseases and Disorders. Biofunctions involved in the RA/CON comparison are presented in orange color, whereas the same biofunctions involved in the COR/RA comparison are presented in green color. -log (*p*-value) > 1.3 represents *p*-value < 0.05.

Based on IPA biofunction analysis, many characteristic proteins of skeletal muscle were differentially expressed following induction of RA and emerged across several highly relevant biofunctions, including “skeletal and muscular system development and function”, “cellular assembly and organization”, and “skeletal muscular disorder” (Figure 7). Of these skeletal muscle proteins, DEPs characteristic of fast-twitch muscle fibers were increased, including parvalbumin (PVALB), fast-type myosin heavy chain 4 (MYH4), fast-type myosin regulatory light chain 2 (MYLPF), and fast-type troponin I (TNNI2), whereas DEPs characteristic of slow-twitch muscle fibers were decreased, including myoglobin (MB), slow-type myosin regulatory light chain 2 (MYL2), slow-type myosin light chain 3 (MYL3), tropomyosin beta chain (TPM2), and slow-type troponin I (TNNI1). Coriander treatment decreased DEPs characteristic of fast-twitch fibers, including PVALB and MYLPF, but increased protein levels of DEPs characteristic of slow-twitch fibers, including MB, MYL2, MYL3, TPM2, and TNNI1 (Table 1).

## 4. Discussion

Currently, there is an increasing interest in exploring the medicinal values of plants as complementary therapies for diseases that have no standard treatments. Coriander, as a widely used spice, vegetable, and folk medicine, has exhibited its various biological functions. As reported, coriander possesses antibacterial [24,25,26], antioxidant [27,28,29], anti-inflammatory [30,31], anticancer [32,33,34], anticonvulsant [35], analgesic [36], and hypoglycemic activities [37,38,39]. Some researchers have even suggested that coriander could be utilized as a complementary therapy or functional food in the treatment of cancer and Alzheimer’s disease [40]. In the current study, we reported the potential use of coriander in the treatment of rheumatoid precachexia.

There is no standard treatment for rheumatoid cachexia. Physical exercise has been proposed as the most effective strategy for mitigating rheumatoid cachexia [5]. However, because RA is commonly observed in elderly individuals, the wide application of physical exercise in the clinical setting is limited. Thus, effective and safe countermeasures against rheumatoid cachexia are urgently needed. During the early stages of cachexia, precachexia is of particular interest because treatment at this stage may prevent and delay the pathogenesis of cachexia [13,14]. However, in contrast to cancer precachexia, rheumatoid precachexia lacks obvious symptoms in patients, making it difficult to recognize and investigate the disease and limiting the discovery of effective pharmaceutical and nutraceutical treatments [8,9].

Abnormal physiological symptoms (swelling of the lower extremities) and biomarkers (increased serum protein levels of TNFα and MMP3) of RA confirmed that our RA rat model was successfully constructed. To carry out our investigation on precachexia, RA rats (only 5 days after disease induction) were chosen as the experimental subjects because significant rheumatoid cachexia has been reported to be observed around 45 days after disease induction in a rat model of RA [12]. Similar to clinical reports of the prevalence of precachexia in patients with RA [9], we did not observe an obvious loss of body weight or appetite in RA rats. However, RA induced a loss of gastrocnemius muscle mass and influenced several biochemical and physiological properties of gastrocnemius muscle within just a few days, as shown in our proteomics study.

RA may cause abnormalities in energy metabolism in the gastrocnemius muscle, resulting in significant attenuation of glycolysis and impaired mitochondrial function, including abnormalities in the TCA cycle and oxidative phosphorylation. Notably, muscle mitochondria dysfunction is associated with neuromuscular junction degeneration, muscle weakness, and muscle atrophy [41,42,43]. Coincidentally, the metabolic derangements and mitochondrial dysfunctions observed in the current study have also been reported in cachectic skeletal muscle under cancer conditions. For example, a significant decrease was observed for proteins participating in glycolysis, the TCA cycle, and the electron transport chain in the cachectic muscles of mice with colon carcinoma [44]. Likewise, there was a reduction of ATP synthesis capacity associated with mitochondrial dysfunction in a lung carcinoma mouse model [45]. The gastrocnemius muscles of RA rats were predicted to be under the energy/ATP-insufficient status because in addition to insufficient energy/ATP production from glycolysis and mitochondria, there was a significant decrease of CKM (cytosolic) and CKMT2 (mitochondrial) proteins, which are involved in the phosphocreatine/creatine (PCr/Cr) shuttle associated with ATP reservation and regeneration [46]. This energy/ATP-insufficient status may contribute to the loss of muscle strength or muscle weakness observed in patients with RA [9]. Coriander treatment restored the levels of various proteins, including GPI, PHDA1, IDH2, ATP5F1A, ATP5F1B, and CKMT2, suggesting that coriander may ameliorate muscle weakness in patients with RA.

RA may cause alterations in myofiber composition in the gastrocnemius muscle. Some proteins characteristic of fast-twitch fibers (e.g., PVALB, MYH4, MYLPF, and TNNI2) were increased, whereas some proteins characteristic of slow-twitch fibers (e.g., MB, MYL2, MYL3, TPM2, and TNNI) were decreased, suggesting an increase in the ratio of fast/slow-twitch fibers in the gastrocnemius muscles of RA rats. This alteration has also been observed in skeletal muscles under conditions such as reduced neuromuscular activity [47], mechanical unloading [48,49], and hyperthyroidism [50], and in patients with chronic inflammation diseases, such as obesity, type II diabetes [51], and cancer cachexia [52]. Myofibers transition in cancer cachectic muscle, shifting from a more oxidative type (slow-twitch fiber) to a more glycolytic type (fast-twitch fiber), and this process has been reported to be associated with mitochondria uncoupling [53]. Notably, skeletal muscle having an increased ratio of fast/slow-twitch fibers may be more vulnerable to atrophy [54]. Therefore, these alterations in myofiber composition may contribute to or be indicative of muscle atrophy and muscle wasting in cachexia. Coriander treatment restored the levels of various proteins, including PVALB, MYLPF, MB, MYL2, MYL3, TPM2, and TNNI, suggesting that coriander may have potential applications in slowing the fiber-type transition.

Within only a few days, RA already caused a disruption of energy metabolism and alterations in myofiber composition in gastrocnemius muscles. These alterations are thought to be associated with muscle atrophy occurring in cancer cachexia and other chronic and/or systemic inflammation diseases. Although, without significant physiological symptoms (e.g., loss of body weight and appetite), a series of alterations at the molecular level indicated that the gastrocnemius muscles of RA rats underwent the transitional process from precachexia to cachexia. We highly recommend that relevant interventions and/or preventive actions, which may ameliorate these metabolic disruptions and alleviate systemic inflammation and oxidative stress, should be applied during early RA onset and/or precachexia rather than waiting until cachexia occurs.

In conclusion, our proteomics study revealed that coriander treatment could improve the physiological and biochemical disorders induced by RA in gastrocnemius muscles. In particular, coriander treatment partially rescued metabolic disruptions in the gastrocnemius muscles by upregulating glycolysis and restoring mitochondria function. Moreover, coriander treatment also partly prevented the physiological abnormalities induced by RA, particularly with regard to myofiber composition. Combining the bio-efficacy of coriander discovered in the current study with the edible and natural properties of coriander, we believe that coriander could be an effective functional food and/or complementary countermeasure for alleviating skeletal muscular disorders and slowing cachexia in patients with RA.

## Figures and Tables

**Table 1:** List of DEPs/proteins related to myofibers.

UniProt ID	Protein Name, Symbol (from IPA)	FC (RA/CON)	FC (COR/RA)
Characteristic proteins of fast-twitch fibers			
P02625	Parvalbumin alpha (PVALB)	1.55	−1.35 ^b^
Q29RW1	Myosin heavy chain 4 (MYH4)	1.98 ^b^	−1.15 ^b^
P04466	Myosin regulatory light chain 2 (fast skeletal muscle isoform) (MYLPF)	1.24	−1.22 ^b^
P27768	Troponin I (fast skeletal muscle) (TNNI2)	1.21	1.01
Characteristic proteins of slow-twitch fibers			
Q9QZ76	Myoglobin (MB)	−1.75 ^(0.07)^	1.27
P08733	Myosin regulatory light chain 2 (slow skeletal/cardiac muscle isoform) (MYL2)	-10 ^a^	4.32 ^(0.09)^
P16409	Myosin light chain 3 (slow-twitch muscle) (MYL3)	−8.33 ^b^	4.25 ^a^
P58775	Tropomyosin beta chain (slow skeletal muscle isoform) (TPM2)	−1.22 ^(0.08)^	1.21
P13413	Troponin I (slow skeletal muscle) (TNNI1)	-5 ^a^	3.11 ^(0.06)^

^a^ *p* < 0.05, ^b^ *p* < 0.01. FC: fold change.

## Data Availability

Not applicable.

## Supplementary Materials

The following are available online at https://www.mdpi.com/article/10.3390/nu13114041/s1, Figure S1: KEGG graph of the citrate cycle (TCA cycle) with fold change information. Figure S2: KEGG graph of glycolysis/gluconeogenesis with fold change information. Figure S3: KEGG graph of oxidative phosphorylation with fold change information. Table S1: Diet composition.
