# Supplementary material for: Ameliorating Effects of Coriander on Gastrocnemius Muscles Undergoing Precachexia in a Rat Model of Rheumatoid Arthritis: A Proteomics Analysis
Source: Nutrients. 2021 Nov 12;13(11):4041. doi: 10.3390/nu13114041 (PMC8621435; doi:10.3390/nu13114041)
Supplement: Supplementary file 1 [file nutrients-13-04041-s001.zip › nutrients-1431967-supplementary.pdf]

Supplementary Figure Legend

Figure S1: KEGG graph of "citrate cycle (TCA cycle)" with DEP identified in comparison RA/CON (left part) and COR/RA (right part). Fold change information is indicated in colors from low (green) to high (red).

Figure S2: KEGG graph of "glycolysis/gluconeogenesis" with DEP identified in comparison RA/CON (left part) and COR/RA (right part). Fold change information is indicated in colors from low (green) to high (red).

Figure S3: KEGG graph of "oxidative phosphorylation" with DEP identified in comparison RA/CON (left part) and COR/RA (right part). Fold change information is indicated in colors from low (green) to high (red).

Figure S1

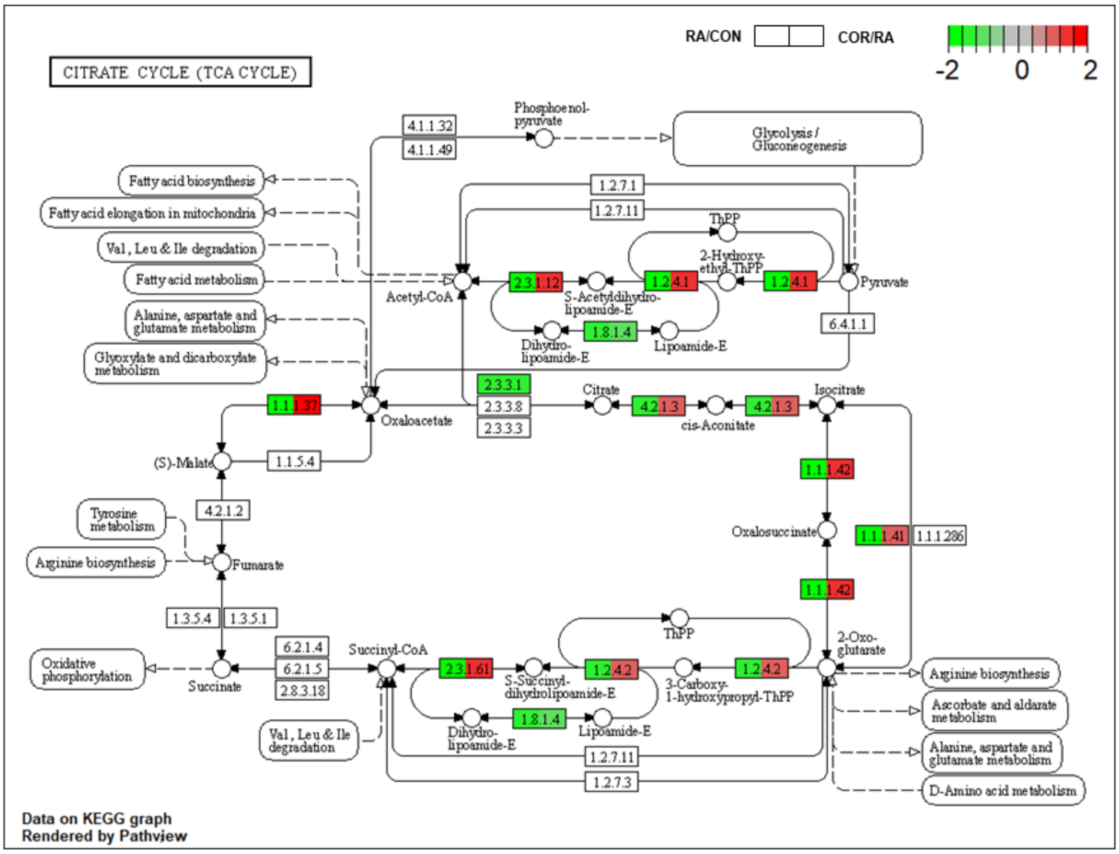
RA/CON
COR/RA
-2
0
2
CITRATE CYCLE (TCA CYCLE)
Phosphoenol-pyruvate
4.1.1.32
4.1.1.49
Glycolysis / Gluconeogenesis
Fatty acid biosynthesis
Fatty acid elongation in mitochondria
Val, Leu & Ile degradation
Fatty acid metabolism
Alanine, aspartate and glutamate metabolism
Glyoxylate and dicarboxylate metabolism
1.2.7.1
1.2.7.11
ThPP
2-Hydroxy-ethyl-ThPP
Acetyl-CoA
2.3.1.12
S-Acetyldihydro-lipoamide-E
1.2.4.1
Pyruvate
1.8.1.4
Dihydro-lipoamide-E
Lipoamide-E
6.4.1.1
2.3.3.1
2.3.3.8
2.3.3.3
Citrate
4.2.1.3
cis-Aconitate
Isocitrate
1.1.1.37
Oxaloacetate
(S)-Malate
1.1.5.4
1.1.1.42
Oxalosuccinate
1.1.1.41
1.1.1.286
4.2.1.2
Tyrosine metabolism
Arginine biosynthesis
Fumarate
1.3.5.4
1.3.5.1
6.2.1.4
6.2.1.5
2.8.3.18
Oxidative phosphorylation
Succinate
Succinyl-CoA
2.3.1.61
S-Succinyl-dihydrolipoamide-E
1.2.4.2
3-Carboxy-1-hydroxypropyl-ThPP
2-Oxo-glutarate
Ascorbate and aldarate metabolism
D-Amino acid metabolism
Val, Leu & Ile degradation
1.2.7.11
1.2.7.3
Data on KEGG graph
Rendered by Pathview

Figure S2

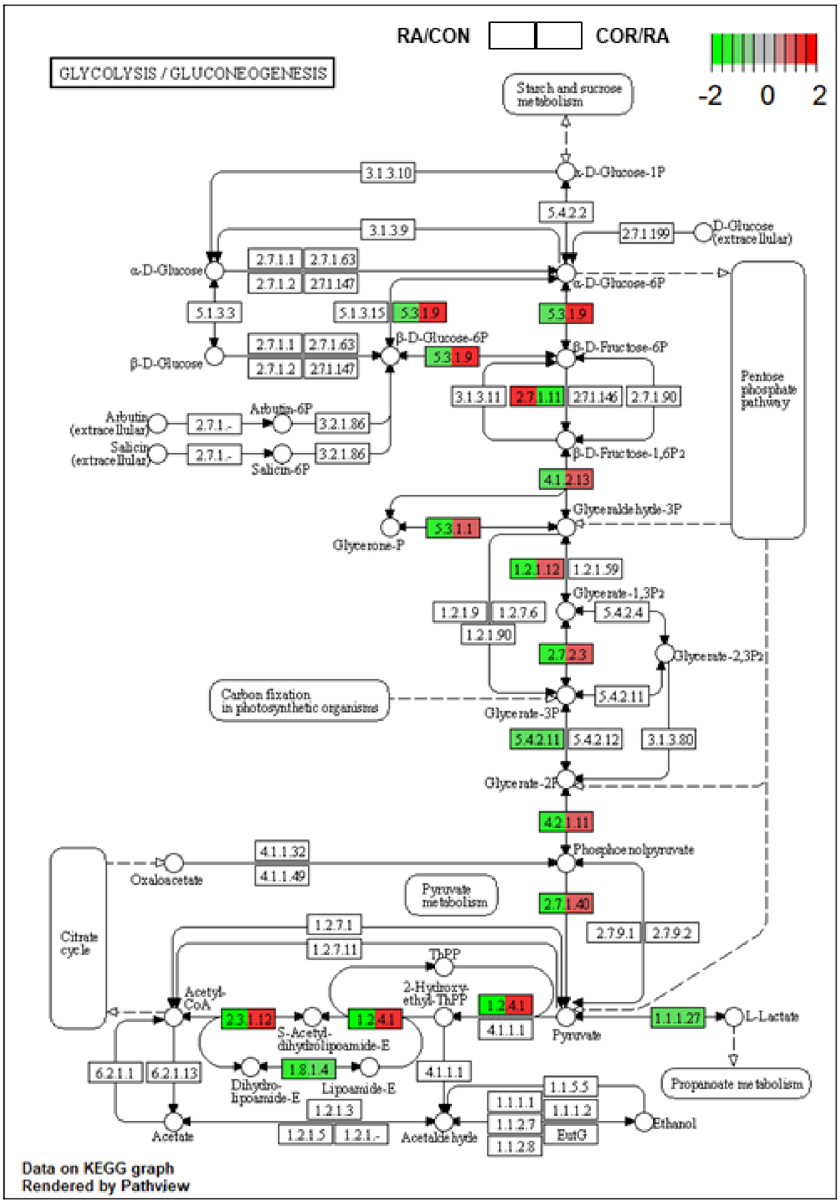

RA/CON
COR/RA
-2
0
2
GLYCOLYSIS / GLUCONEOGENESIS
Starch and sucrose metabolism
α-D-Glucose-1P
3.1.3.10
5.4.2.2
3.1.3.9
2.7.1.199
D-Glucose (extracellular)
α-D-Glucose
2.7.1.1
2.7.1.63
2.7.1.2
2.7.1.147
α-D-Glucose-6P
5.1.3.3
5.1.3.15
5.3.1.9
β-D-Glucose-6P
β-D-Fructose-6P
β-D-Glucose
Pentose phosphate pathway
3.1.3.11
2.7.1.11
2.7.1.146
2.7.1.90
Arbutin (extracellular)
2.7.1.-
Arbutin-6P
3.2.1.86
Salicin (extracellular)
Salicin-6P
β-D-Fructose-1,6P2
4.1.2.13
Glyceraldehyde-3P
5.3.1.1
Glycerone-P
1.2.1.12
1.2.1.59
Glycerate-1,3P2
1.2.1.9
1.2.7.6
5.4.2.4
1.2.1.90
2.7.2.3
Glycerate-2,3P2
Carbon fixation in photosynthetic organisms
5.4.2.11
Glycerate-3P
5.4.2.12
3.1.3.80
Glycerate-2P
4.2.1.11
4.1.1.32
Phosphoenolpyruvate
Oxaloacetate
4.1.1.49
Pyruvate metabolism
2.7.1.40
Citrate cycle
1.2.7.1
1.2.7.11
2.7.9.1
2.7.9.2
ThPP
2-Hydroxy-ethyl-ThPP
Acetyl-CoA
2.3.1.12
1.2.4.1
S-Acetyl-dihydrolipoamide-E
4.1.1.1
Pyruvate
1.1.1.27
L-Lactate
6.2.1.1
6.2.1.13
1.8.1.4
Lipoamide-E
Dihydro-lipoamide-E
1.1.5.5
Propanoate metabolism
1.1.1.1
1.1.1.2
1.2.1.3
1.1.2.7
Ethanol
1.2.1.5
1.2.1.-
EutG
Acetate
Acetaldehyde
1.1.2.8
Data on KEGG graph
Rendered by Pathview

Figure S3

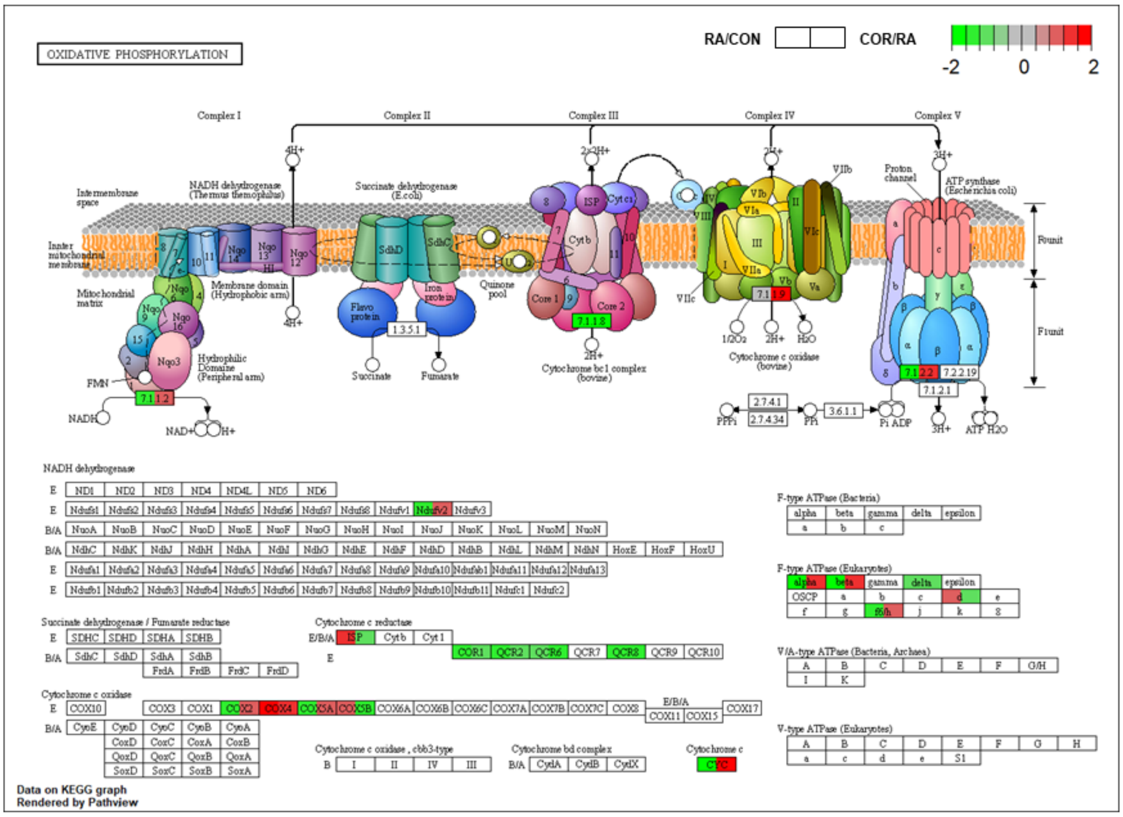
OXIDATIVE PHOSPHORYLATION
RA/CON
COR/RA
-2
0
2
Complex I
Complex II
Complex III
Complex IV
Complex V
NADH dehydrogenase
Succinate dehydrogenase / Fumarate reductase
Cytochrome c reductase
Cytochrome c oxidase
F-type ATPase (Bacteria)
F-type ATPase (Eukaryotes)
V/A-type ATPase (Bacteria, Archaea)
V-type ATPase (Eukaryotes)
Data on KEGG graph
Rendered by Pathview

Table S1. Composition of experimental diets

Ingredient	AIN-93G diet (% w/w)	COR diet (% w/w)
**Cornstarch**	**39.7**	**37.7**
**Casein**	**20**	**20**
**Dextrinized cornstarch**	**13.2**	**13.2**
**Sucrose**	**10.3**	**10.3**
**Soybean oil**	**7**	**7**
**Fiber**	**5**	**5**
**Mineral mix (AIN 93G-MX)**	**3.5**	**3.5**
**Vitamin mix (AIN 93G-MX)**	**1**	**1**
**L-Cystine**	**0.3**	**0.3**
**Coriander**	**0**	**2**
**Eggshell membrane**	**0**	**0**
